# Bacteria slingshot more on soft surfaces

**DOI:** 10.1038/ncomms6541

**Published:** 2014-11-21

**Authors:** Rongrong Zhang, Lei Ni, Zhenyu Jin, Jiahong Li, Fan Jin

**Affiliations:** 1 Hefei National Laboratory for Physical Sciences at the Microscale, University of Science and Technology of China, Hefei 230026, China; 2 Department of Chemical Physics, University of Science and Technology of China, Hefei 230026, China; 3 Department of Polymer Science and Engineering, University of Science and Technology of China, Hefei 230026, China; 4 CAS Key Laboratory of Soft Matter Chemistry, University of Science and Technology of China, Hefei 230026, China

## Abstract

Adaptive responses greatly improve the competitive capacities of bacteria in diverse environments. Here, we investigate whether bacteria can adapt to a microenvironment with distinctive softness by examining the type-IV pili (TFP)-mediated motility of *Pseudomonas aeruginosa* cells on brush-like surfaces that are grafted with a layer of thermally sensitive polymer chains, where the softness of the brush-layer is tunable by applying a small temperature change (from 30 to 37 °C). We report that *P. aeruginosa* cells slingshot more on soft surfaces at a shear-thinning condition, which greatly facilitates their surface crawling by means of reducing energy dissipation. This adaptive response suggests that *P. aeruginosa* cells may be able to sense the local viscoelasticity and then deploy TFP to adapt to their physical surroundings.

## Abstract (web summary)

Pseudomonas aeruginosa migrate by deploying their type-IV pili. Here, Zhang *et al*. show that *P. aeruginosa* can adapt to the physical microenvironment by using their type-IV pili to ‘slingshot’ more across soft surfaces to exploit inherent shear thinning properties of the surface.

## Graphical abstract

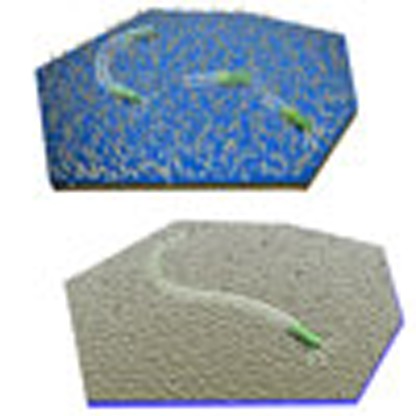

Over 99% of all bacteria live in biofilms that are multicellular surface-associated communities that appear ubiquitously in environmental, technological and medical settings^1^,^2^,^3^. The formation of gram-negative bacterial biofilms is facilitated by surface motility modes in the initial stages, where planktonic bacteria first become surface-associated and subsequently self-assemble into microcolonies^4^,^5^. Twitching motility, a well-known surface motility mode, mediated by type-IV pili (TFP)^6^,^7^, is employed by many bacterial species including the opportunistic pathogen *Pseudomonas aeruginosa*^8^, the gonorrheal bacterium *Neisseria gonorrhoeae*^9^ and the predatory soil bacterium *Myxococcus xanthus*^10^. It has been known that twitching plays an essential role for these and other species of bacteria, enabling them to colonize abiotic/biotic surfaces and to form bacterial biofilms^6^ that, on human tissues or medical settings, may lead to harmful or fatal infections^1^,^11^. Recently, a number of investigations^12^,^13^ have shown that bacteria can use their TFP to walk or crawl on surfaces, in which walking or crawling motility can mediate cells to spread on surfaces with distinctive trajectories by means of short or long persistence lengths. Moreover, Jin *et al*.^14^ have shown that bacteria can differently deploy their TFP to alternatively generate pull actions and slingshots in the crawling motility, where the velocities of slingshots are 20 times higher than those of pull actions. An intriguing question suggested by the aforementioned findings is whether bacteria can differentially deploy their TFP to generate proper motility types in response to different environmental cues.

Bacteria are thought to be able to adapt TFP-mediated motility in response to nutrient availability or chemical cues^15^ because the growth^16^, extension and retraction of TFP are tightly regulated by chemotactic and cyclic-bis-(3′-5′)-dimeric-GMP (c-di-GMP) signalling pathways^8^,^17^. Aside from chemical cues, different abiotic/biotic surfaces often possess distinctive physical properties, such as viscoelasticity; for example, there is a shear modulus difference of at least six magnitudes between tissues and alloyed bone nail surfaces, yet bacterial biofilms can form on both^18^. However, it is less certain whether, especially at the single-cell scale, bacteria can adapt TFP-mediated motility or other surface motilities in response to their physical surroundings; only a few investigations suggest that bacteria do so^4^,^19^,^20^. Answering the question of whether bacteria can adapt TFP-mediated motility in response to specific physical cues, aside from answering a fundamental question in biophysics, has implications for ongoing research in seeking applicable biomaterials to prevent biofilm formation on medical settings. Here, we directly answer this question by investigating how individual *P. aeruginosa* cells deploy their TFP to crawl on a polymeric surface with a tunable softness. After carefully excluding other possible factors, we demonstrate that bacteria deploy their TFP to slingshot more on soft surfaces at a shear-thinning condition. Moreover, we find that this response facilitates cells to disperse on surfaces by means of reducing energy dissipation.

## Results

### Smart polymeric surfaces with a tunable softness

We grafted thermally sensitive poly(*N*-isopropylacrylamide) (PNIPAAM) chains on coverslips by surface-initiated atom transfer radical polymerization (SI-ATRP). SI-ATRP is a well-developed method that can be used to homogenously graft polymer chains on a surface with a high grafting density^21^. The synthetic methods are given in Methods and in Supplementary Fig. 1. It is well-known that adjusting the environmental temperature (*T*) around the transition point (*T*_c_≃32 °C) changes the conformation of PNIPAAM chains greatly on surfaces and in solutions^22^,^23^. In the present study, raising *T* from lower (*T*<*T*_c_) to higher (*T*>*T*_c_) leads to the dehydration of brushy chains on the surface; in this transition, a fluid-like polymeric layer will collapse onto a surface to form a solid-like layer. By using a quartz crystal microbalance^22^,^24^, we confirmed that this transition does occur, resulting in a significantly increased hardness of surface (additional details are given in Supplementary Methods and in Supplementary Fig. 2). Moreover, the dehydration of PNIPAAM chains on surfaces can be monitored by their water-contacting angles (*θ*_w_) because this process increases surface hydrophobicity^25^. Supplementary Fig. 3f,g shows that dehydration occurs at the range of 31 °C<*T*<36 °C, which is consistent with existing literature^22^. Taken together, PNIPAAM brush surfaces can be tuned from soft to hard by only a small temperature change from 30 to 37 °C.

Below the *T*_c_ (*T*=30 °C), fluid-like grafting chains can either stay at ‘mushroom-like’ or at ‘brush-like’ conformations on surfaces^26^, which is determined by their 
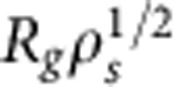
, where *R*_g_ is the radius of gyration of grafting chains at an unperturbed condition, *ρ*_s_ is the number density of grafting chains at surface. Table 1 indicates that our prepared PNIPAAM surfaces possess a sufficiently high *ρ*_s_ 
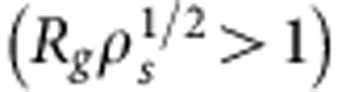
, which enforces those grafting chains to stay at ‘brush-like’ conformations. As the static viscosity (*η*) and the relaxation time (*τ*) of polymer brushes depend on the number average molar mass (*M*_n_) of grafting chains, we thereby prepared two distinctive PNIPAAM surfaces to control *η* and *τ*, on which the grafting chains possess distinctive *M*_n_, that is, *M*_n_=12,800 or *M*_n_=138,000; we denote them below as short-brush or long-brush surfaces, respectively. Next, we estimated *η* and *τ* of short-brush or long-brush surfaces (see Methods and Table 1). We found that the major difference between short- and long-brush surfaces is their relaxation time, which is due to 
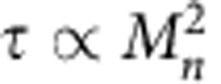
. Finally, we examined whether these PNIPAAM brush surfaces are compatible to *P. aeruginosa* by investigating the growth of cells on long-brush surfaces at 30 and 37 °C, respectively. Our results show normal bacterial growth and subsequent nascent biofilm formation on PNIPAAM brush surfaces, as shown in Supplementary Fig. 4, thus demonstrating that these brush surfaces are non-toxic for *P. aeruginosa*.

### Tracking the crawling motility in *P. aeruginosa*

**Figure 1:**
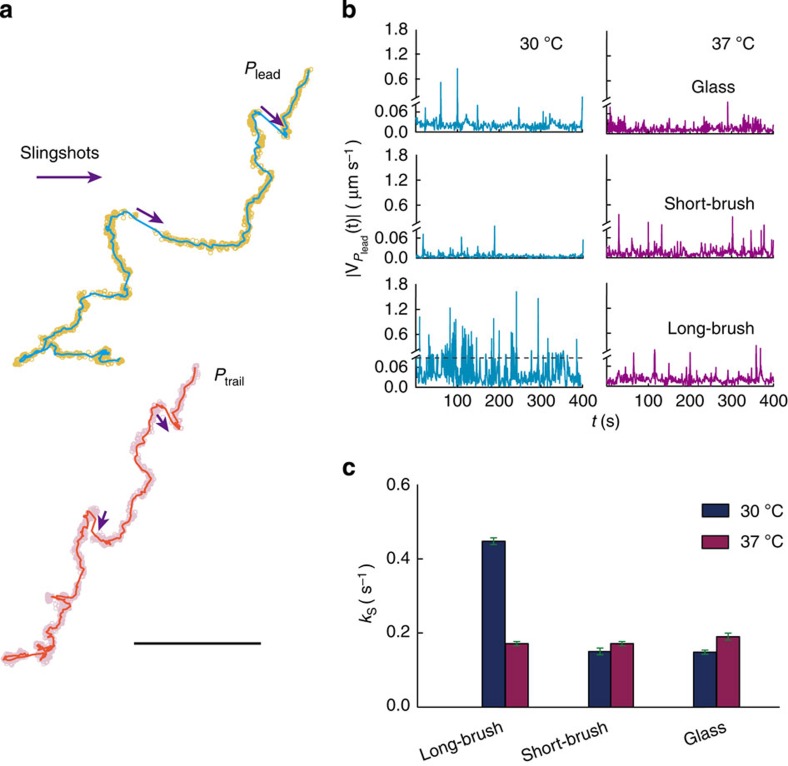
Slingshot of *P. aeruginosa* cells on PNIPAAM surfaces. (**a**) Two-points tracking of a representative trajectory from a crawling *P. aeruginosa*, where symbols or lines represent the original or the denoised data, respectively, the scale bar, 1 μm. (**b**) Representative instantaneous velocities 
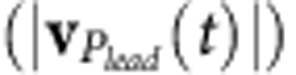
 or (**c**) rate of slingshots (*k*_s_) in crawling cells at different conditions, where the dash line in (**b**) represents the threshold that was used to detect slingshots, the average rate of slingshots in each condition arose from the analysis of multiple cells (35 to 77), the results obtained from multiple technical replicates (≥3) and the error bar represents a s.d.

To isolate TFP-mediated motilities in *P. aeruginosa*^12^,^13^, we monitored the crawling of flagellum-deficient cells (Δ*fliM*) in various surface conditions using bright-field microscopy with high temporal-spatial resolutions. Next, we analysed the motion of those cells by using a two-point tracking algorithm that can separately track the trajectories (**r**_lead_(*t*), **r**_trail_(*t*)) of leading (*P*_lead_) and trailing poles (*P*_trail_) for every individual cell over time^14^. We have previously shown that sub-pixel noises (0.5) inherently exist in those original trajectories^14^. To gain higher temporal-spatial resolutions, we denoised those trajectories using a wavelet set of Daubechies that can significantly suppress the noises^27^, as shown in Fig. 1a. Thus, we can resolve instantaneous velocities 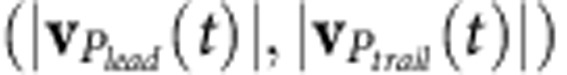 of *P*_lead_ and *P*_trail_ ranging from 0.01 to 3 μm s^−1^. As we have previously shown^14^, two distinctive features can be found in typical **r**_lead_(*t*), as shown in Fig. 1a, namely, (1) a continuous positioning of **r**_lead_(*t*), reflecting low-speed motions, the so-called pulling action and (2) a discontinuous positioning of **r**_lead_(*t*), denoting high-speed motions, the so-called slingshot with a much greater velocity than that of the pulling action (Supplementary Fig. 5). It is this greater velocity that enables us to detect the slingshot in **r**_lead_(*t*) by a threshold (0.09 μm s^−1^). To further confirm that the pulling action and slingshot are mediated by TFP exclusively, we investigated the surface motilities of two additional mutants, Δ*rhlA*Δ*fliM* and Δ*pilA*Δ*fliM*. Δ*rhlA*Δ*fliM* is deficient in producing rhamnolipids^28^ and lacks flagellum; Δ*pilA*Δ*fliM* lacks both the motility appendages of TFP and flagellum. Supplementary Fig. 5a clearly indicates that the pulling action and slingshot in the crawling motility are strictly mediated by TFP.

### Slingshot on polymeric surfaces with distinctive softness

First, we investigated the crawling of cells on long-brush surfaces. We surprisingly observed that cells like to slingshot more on this surface at 30 °C (Supplementary Movie 1) in comparison with those at 37 °C (Supplementary Movie 2), as shown in Fig. 1b. We found the rate of slingshot (*k*_s_) increases ~3-fold by measuring multiple cells, as shown in Fig. 1c. Aside from the softness of surfaces, a number of possible factors may result in the increase of *k*_s_, including (1) a change of environmental temperature; (2) the chemical modification of the surface by PNIPAAM chains; (3) a change of surface hydrophobicity; (4) the alteration of the interaction between bacteria and surfaces and (5) the surface heterogeneity. To rule out the first possibility, we repeated the experiments on unmodified coverslips and the results showed that increasing *T* from 30 to 37 °C does not alter *k*_s_, as shown in Fig. 1b,c. To address the second possibility, we investigated the crawling of cells on short-brush surfaces because chemically they are identical with long-brush surfaces. Our results for short-brush surfaces reveal that *k*_s_ remains at the control level determined from unmodified coverslips at 30 or at 37 °C (Fig. 1b,c). However, we also noticed that raising *T* from lower (*T*<*T*_c_) to higher (*T*>*T*_c_) increases hydrophobicity on short-brush surfaces (which it also does almost identically for long-brush surfaces, see Supplementary Fig. 3f,g). Therefore, no increase of *k*_s_ on short-brush surfaces can be used to exclude the third possibility.

To further address this point, we investigated the crawling of cells on hydrophobic glass surfaces. Supplementary Fig. 6 displays that *k*_s_ remains at the control level. To address the fourth possibility, we evaluated the interaction between bacteria and surfaces using surface-shear stress assays^29^. Supplementary Fig. 7 shows that the interaction between bacteria and short-brush surfaces is similar to those of long-brush surfaces at corresponding temperatures of 30 and 37 °C. As for the fifth possibility, we labelled long-brush surfaces using two different fluorescent dyes. Our results indicate that long-brush surfaces are homogenous by means of the spatial localization of grafting chains and the local hydrophobicity, as shown in Supplementary Fig. 3a–c. After excluding all the aforementioned factors, we conclusively demonstrate that *P. aeruginosa* cells like to slingshot more if they meet a surface grafted with long polymer chains. (All additional and relevant details to the above points are given in Supplementary Information.)

### Trajectories on polymeric surfaces with distinctive softness

**Figure 2:**
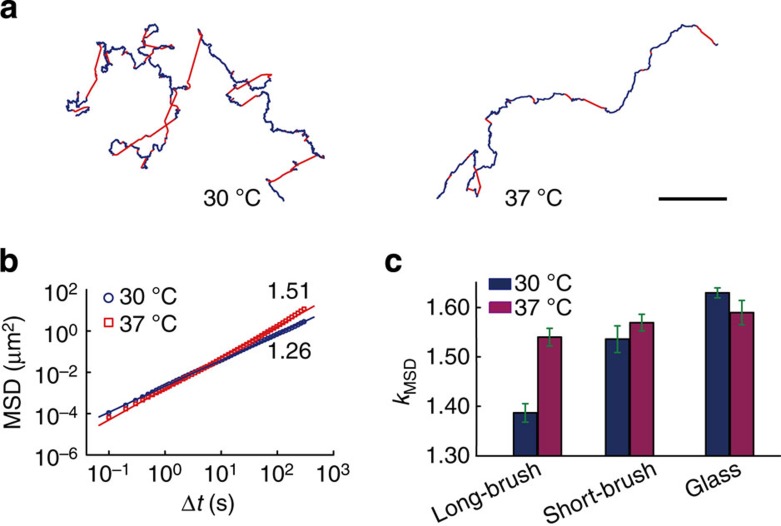
Crawling trajectories of *P. aeruginosa* cells on PNIPAAM surfaces. (**a**) Representative trajectories or (**b**) corresponded mean square displacements (MSD) of crawling cells on a long-brush surface at 30 °C and at 37 °C, where the scale bar in (**a**) is 1 μm. (**c**) Slope of MSD (*k*_MSD_) at different conditions, where the average value of *k*_MSD_ in each condition arises from the analysis of multiple cells (35 to 77), the results obtained from multiple technical replicates (≥3) and the error bar represents a s.d.

Figure 2a displays the typical trajectories of a crawling cell on a long-brush surface at 30 °C and also at 37 °C, where red and blue colours represent slingshots or pulling actions, respectively. We noticed that when compared with the pulling action, the slingshot is more likely to change the direction of crawling. To quantify the directionality of crawling trajectories, we calculated the mean square displacements (MSDs) as a function of time intervals (Δ*t*), and extracted their slope (*k*_MSD_) by linear fits of log(MSD) as a function of log(Δ*t*), that is, log(MSD)∝*k*_*MSD*_log(Δ*t*). *k*_MSD_=1.0 corresponds to random diffusive motion without a certain direction, whereas *k*_MSD_=2.0 corresponds to unidirectional ballistic motion. Figure 2b clearly indicates that raising *T* from 30 to 37 °C leads to an increase of *k*_MSD_ from 1.26 to 1.51 on long-brush surfaces, indicating that the crawling of the cell becomes more directional. To further confirm this result, we analysed multiple crawling trajectories on different surface conditions, as shown in Fig. 2c. Our results explicitly indicate that *k*_MSD_ is smaller on long-brush surfaces at 30 °C when compared with those that arise from control experiments (Fig. 2c and Supplementary Fig. 8).

## Discussion

**Figure 3:**
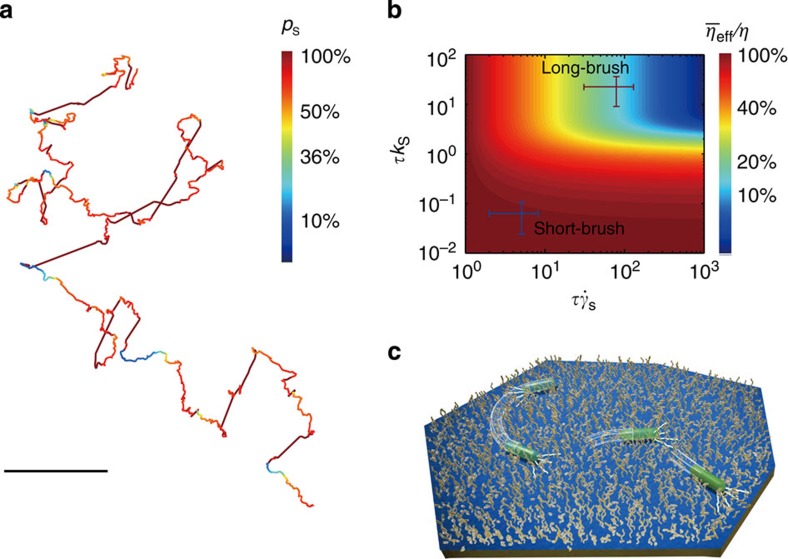
Slingshot of *P. aeruginosa* cells on soft surfaces under a shear-thinning condition. (**a**) Representative trajectory of a crawling cell on long-brush surfaces at 30 °C, where colours represent magnitudes of the probability (*p*_s_) that grafting chains stay at a shear-thinning condition after being sheared by one slingshot, *τ*=50 s was used here, the scale bar, 1 μm. (**b**) Average effective viscosities 
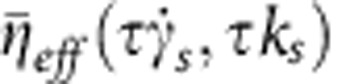
 of the brush-layer as a function of 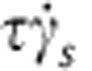 and *τk*_*s*_, where colours represent the magnitudes of 
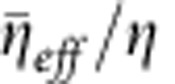
. (**c**) Schematic showing that bacterium deploys TFP to slingshot more on a soft surface.

Our results indicate that bacteria only slingshot more on long-brush surfaces than on short-brush surfaces at 30 °C. The major difference in these surfaces is their relaxation time (*τ*_short_=0.1~1 s and *τ*_long_=10~100 s), which prompts the question of why bacteria like to slingshot more on a soft surface that has a longer relaxation time. We estimated the shear rate of slingshot 
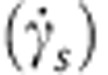
 on short- or long-brush surfaces using a relation 
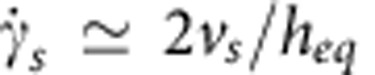
, where *v*_s_ is the averaged velocity of slingshots and *h*_eq_ is the equilibrium height of the brush-layer. We found that 
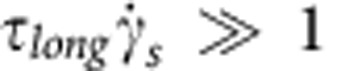
, but 
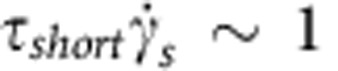
, indicating that slingshot on long-brush surfaces can lead the brush-layer to tune into the condition of shear-thinning. Therefore, one can expect that long-brush chains cannot relax during the *τ*_long_ after being sheared by a slingshot, which can markedly reduce the effective viscosity (*η*_eff_) following 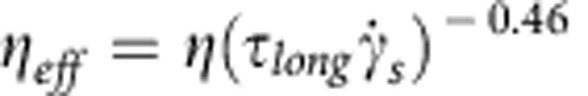 (refs 30, 31). Note that the shear-thinning affects the low-speed pulling action in the duration of *τ*_long_ because the displacement of single pulling actions or slingshots is relatively smaller (Fig. 2a) in comparison with the bacterial length. To account the overall shear-thinning effect, we calculated the time-average effective viscosity 
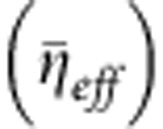
 as


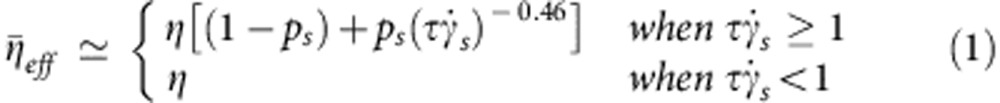


where *p*_s_ is the probability that the brush-layer stays at the shear-thinning condition. One can expect that 
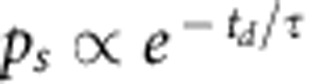
, where *t*_d_ is the time delay after one slingshot, as shown in Fig. 3a. The average value of *t*_d_ can be estimated by ~1/*k*_s_ so that when *τk*_s_>>1, equation (1) can be rewritten as


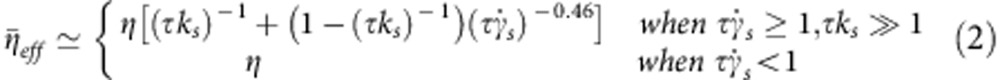


A two-dimensional plotting of 
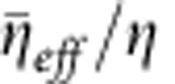
 as the function of 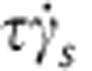 and *τk*_*s*_ is shown in Fig. 3b, where colours represent the magnitude of 
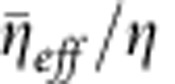
. It clearly indicates that at the regimes of 
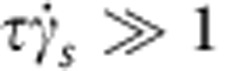
and *τk*_*s*_>>1, 
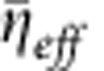
 is greatly reduced; for example, frequent slingshot (*τk*_*s*_>>1) on the long-brush surface 
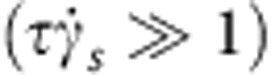
 can reduce energy dissipations up to 90%. Therefore, this TFP-mediated response can facilitate bacteria crawling on surfaces. Considering that *P. aeruginosa* continuously secretes extracellular polymeric substances (EPS) to form biofilms^32^, we also expect that cells will slingshot even more on a rigid surface if it is covered by secreted EPS, as both the viscosity and relaxation time of EPS^33^ is comparable to those of long-brush surfaces.

In summary, we have conclusively demonstrated that *P. aeruginosa* cells slingshot more on a soft surface when 
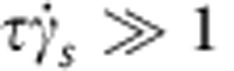
, which can greatly reduce surface viscosity and thus facilitate surface crawling. This adaptive response of *P. aeruginosa* suggests that those cells may be able to sense their local viscoelasticity and subsequently deploy their TFP to adapt to their surroundings. Figure 3c graphically summarizes our findings. In the real world, this type of adaptive bacteria behaviour may be observed on medical settings coated with a polymeric layer or even on human tissues. The deployment of TFP to slingshot can lead bacteria to spread and colonize on soft surfaces at a shear-thinning condition, hence facilitating biofilm formation. Therefore, this fundamental finding has implications for a number of research fields and may also provide an applicable approach for interfering with TFP-mediated motilities and any subsequent biofilm formation by controlling the surface softness of materials on which bacterial biofilms may form.

## Methods

### Materials

The coverslips (24 × 60 × 0.17 mm^3^, Fisher) were pre-washed by sonication (5 min) in acetone, ethanol and ultrapure water (18.2 MΩ cm, Millipore), respectively, and rinsed with 5% (aq.) hydrogen fluoride for 10 s, and then washed in water immediately. These coverslips were finally dried with nitrogen and further treated (5 min) with a plasma cleaner before use. After wash, the water contact angles on these coverslips approach 0°. The monomer (*N*-isopropyl-acrylamide, Aladdin) was recrystallized (× 3) in toluene/hexane (35:65, v-v) before use. Solvents, including toluene and dimethyl sulfoxide (DMSO), were purified according to the standard protocols. The [(chloromethyl)phenylethyl]- Trimethoxysilane (CTMS), Methyl 4-(chloromethyl) benzoate were purchased from Gelest and Tci, respectively and used without further purification. Tris[2-(dimethylamino)ethyl]- amine (Me_6_TREN) was prepared according to the previous literature^34^.

### Preparation of PNIPAAM brush surfaces

The SI-ATRP was used to graft the polymer chains from surfaces^21^. First, the initiators were linked to the coverslips by refluxing CTMS in dry toluene at 125 °C for 4 h, and then monomers (NIPAAM) were polymerized from initiator-modified coverslips in a DMSO solution containing free initiator (Methyl 4-(chloromethyl) benzoate), ligand (Me_6_TREN) and catalyst (cuprous chloride). This reaction was allowed to proceed at room temperature over 24 h and was terminated by inducting air. Note that the monomers were simultaneously polymerized in the solution. Thus, after the polymerization, the *M*_n_ and the polydispersity indexes (PD.I.) of polymer chains in the solution are expected to be comparable to those chains grafted on the surface. Previous investigations have suggested that this estimation would lead an uncertainty of ±25% (refs 35, 36). The above reactions were summarized in Supplementary Fig. 1a. The resultant coverslips were washed with fresh DMSO and sodium EDTA (aq.) and then dried with nitrogen before use. *M*_n_ can be controlled by adjusting the mole ratio of monomer to initiator. The hydrophobic glass surfaces were prepared by coupling *n*-Dodecyltrimethoxysilane to coverslips in dry toluene.

### Characterization of PNIPAAM brush surfaces

We first examined the PNIPAAM brush surfaces using X-ray photoelectron spectroscopy (XPS) and found the nitric peak appearing in XPS spectrum, as shown in Supplementary Fig. 1b, suggesting that PNIPAAM chains were successfully grafted on the coverslips. By using gel permeation chromatography (GPC), we next estimated *M*_n_ and PD.I. of the grafting chains by characterizing those free chains that were polymerized on the solutions. GPC results display that the PD.I. of those free polymer chains are <1.3, as shown in Supplementary Fig. 1c, which suggest that the molar mass distributions of those grafting chains are narrow. The dry thickness (*h*_dry_) of the film formed by the PNIPAAM brush surfaces was characterized using an ellipsometry (M-2000V-ESM, J.A. Woollam Co.), in which translucent adhesive tapes (Scotch 810 Magic Tape, 3 M) were stuck on the back-surfaces to eliminate backside reflections. Afterwards, *ρ*_s_ was determined by *ρ*_s_=*h*_dry_*ρ*_f_*N*_A_/*M*_n_ (ref. 37), where *ρ*_f_ is the density of PNIPAAM dry films and *N*_A_ is the Avogadro’s number. Finally, we measured the contact angles (*θ*_w_) of a FAB droplet on short- or on long-brush surfaces at different temperatures, as is shown in Supplementary Fig. 3f,g. Our results clearly indicate that raising *T* from 31 to 36 °C leads to the increasing of surface hydrophobicity on these surfaces, further demonstrating that PNIPAAM chains were successfully grafted on the coverslips.

### Viscosities and relaxation times of PNIPAAM brush surfaces

In the regime of 
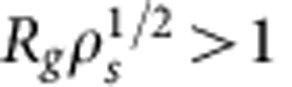
, the equilibrium height (*h*_eq_) of brush-layer can be calculated by *h*_eq_=*N*_p_(2*a*^5^*ρ*_s_/3)^1/3^ (ref. 26), where *a* (=0.3 nm) is the monomer size^38^. Knowing the concentration (*c*=*ρ*_s_*M*_n_/*N*_A_*h*_eq_) of grafting chains in the brush layer, one can calculate *η* using the intrinsic viscosities ([*η*]) of grafting chains^39^,^40^. It has been known that the relaxation of grafting chains in polymer brushes is comparable to the relaxation of arms in star polymers^41^,^42^. Therefore, we estimated *τ* of PNIPAAM brush surfaces using the relaxation time arising from star polymers that have comparable polymer concentrations and chain lengths. Note that relative errors in the estimation of *τ* are around one magnitude. Additional details for the estimation of *τ* are given in Supplementary Note 1.

### Bacterial strains and flow cell experiments

Three mutants of *P. aeruginosa* (ATCC 15692), Δ*fliM*, Δ*rhlA*Δ*fliM* and Δ*pilA*Δ*fliM*, were used in this study, as is shown in Supplementary Table 1. Strains were grown on LB agar plates at 37 °C for 24 h. Monoclonal colonies were inoculated into 5 ml polystyrene culturing tubes containing 1 ml of minimal medium (FAB)^43^ and then grown in a shaker at 37 °C, in which 30 mM glutamate was added as the carbon source. Bacterial cultures were harvested at exponential phase, as monitored by their optical densities at 600 nm (OD_600_≈0.8). The resultant cultures were further diluted by adding 30 μl of the bacterial suspension into 1,000 μl of sterilized FAB. Flow cells (Denmark Technical University) used here were prepared and sterilized under the standard protocol. After an injection of bacterial cultures, the flow cell was typically given 15 min to allow for the cells to attach to the coverslip, any unattached cells were washed out afterwards. These surface-associated cells were further cultured either at 30.0±0.1 °C or at 37.0±0.1 °C by flowing the FAB with 0.6 mM glutamate with a constant flow rate (3.0 ml h^−1^). Details for the construction of Δ*rhlA*Δ*fliM* and Δ*pilA*Δ*fliM* are given in Supplementary Methods.

### Microscopy and tracking of crawling cells

An Olympus IX81 inverted-microscope equipped with a × 100 oil objective and an sCMOS camera (Neo, Andor) was used to acquire the bright-field images (528 × 512 pixels) of surface-associated cells with a frame rate of 10 fps. A typical movie contains ~18,000 images. Seventy-five movies were acquired and analysed in total, in which the 16-bit greyscale images were first converted to binary images for the detection of bacteria with a standard image processing algorithm, and the x-y positions of leading and trailing poles in single cells were second determined and linked individually over time by using our established two-point tracking algorithm^14^. The resultant trajectories of leading or trailing poles were further denoised using Daubechies wavelets (Fig. 1a)^27^. A velocity threshold (0.09 μm s^−1^) was used to detect the slingshot from the time series of instantaneous velocity.

## Author contributions

F.J. designed the experiments. R.Z. and L.N. performed experiments. R.Z., L.N. and Z.J. analysed data. J.L. helped to prepare PNIPAAM brush surfaces. R.Z. and F.J. contributed jointly to data interpretation and manuscript preparation. All authors reviewed the manuscript.

## Additional information

**How to cite this article:** Zhang, R. *et al*. Bacteria slingshot more on soft surfaces. *Nat. Commun.* 5:5541 doi: 10.1038/ncomms6541 (2014).

## Supplementary Material

Supplementary Information Supplementary Figures 1-8, Supplementary Table 1, Supplementary Note 1, Supplementary Methods and Supplementary References.

Supplementary Movie 1 Crawling of a Δ*fliM* cell on a long-brush surface at30°C, wher blue or red lines represent pulling actions or slingshots respectively.

Supplementary Movie 2 Crawling of a Δ*fliM* cell on a long-brush surface at37°C, wher blue or red lines represent pulling actions or slingshots respectively.

## Figures and Tables

**Table 1:** Characterization of PNIPAAM brush surfaces.

	***M***_**n**_** (g** **mol**^**−1**^**)**	**PD.I.**	***h***_**dry**_ **(nm)**	***ρ***_**s**_ **(nm**^**−2**^**)**
Short-brush	12,800*	1.24	13	0.78
Long-brush	138,000*	1.28	83	0.46
	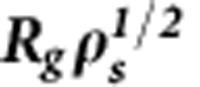	***h***_**eq**_ **(nm)**	***η*** **(P**_**a**_**·s)**	***τ*** **(s)**
Short-brush	3.5	20	0.1	0.1–1
Long-brush	10.1	175	0.3	10–100

Characteristic parameters for short or long-brush PNIPAAM surfaces, including number average mole mass (*M*_n_), polydispersity indexes (PD.I.), dry thickness (*h*_dry_) of the polymeric film, number densities of the grafting chains on surfaces (*ρ*_s_), 
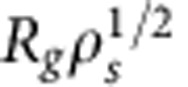
, equilibrium heights (*h*_eq_) of the brush-layers, static viscosities (*η*) and longest relaxation times (*τ*), where 
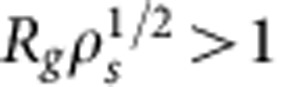
 represents the grafting chains staying at a ‘brush-like’ confirmation, the superscript of ‘*’ represents an uncertainty of ±25%.

## References

[b1] Donlan R. M. & Costerton J. W. Biofilms: Survival mechanisms of clinically relevant microorganisms. Clin. Microbiol. Rev. 15, 167–193 (2002). 11932229 10.1128/CMR.15.2.167-193.2002 PMC118068

[b2] Hall-Stoodley L., Costerton J. W. & Stoodley P. Bacterial biofilms: from the natural environment to infectious diseases. Nat. Rev. Microbiol. 2, 95–108 (2004). 15040259 10.1038/nrmicro821

[b3] Costerton J. W., Stewart P. S. & Greenberg E. P. Bacterial biofilms: a common cause of persistent infections. Science 284, 1318–1322 (1999). 10334980 10.1126/science.284.5418.1318

[b4] O'Toole G. A. & Kolter R. Flagellar and twitching motility are necessary for Pseudomonas aeruginosa biofilm development. Mol. Microbiol. 30, 295–304 (1998). 9791175 10.1046/j.1365-2958.1998.01062.x

[b5] Klausen M., Aaes-Jorgensen A., Molin S. & Tolker-Nielsen T. Involvement of bacterial migration in the development of complex multicellular structures in Pseudomonas aeruginosa biofilms. Mol. Microbiol. 50, 61–68 (2003). 14507363 10.1046/j.1365-2958.2003.03677.x

[b6] Harshey R. M. Bacterial motility on a surface: Many ways to a common goal. Annu. Rev. Microbiol. 57, 249–273 (2003). 14527279 10.1146/annurev.micro.57.030502.091014

[b7] Mattick J. S. Type IV pili and twitching motility. Annu. Rev. Microbiol. 56, 289–314 (2002). 12142488 10.1146/annurev.micro.56.012302.160938

[b8] Burrows L. L. *Pseudomonas aeruginosa* twitching motility: Type IV pili in action. Annu. Rev. Microbiol. 66, 493–520 (2012). 22746331 10.1146/annurev-micro-092611-150055

[b9] Maier B., Potter L., So M., Seifert H. S. & Sheetz M. P. Single pilus motor forces exceed 100 pN. Proc. Natl Acad. Sci. USA 99, 16012–16017 (2002). 12446837 10.1073/pnas.242523299 PMC138556

[b10] Sun H., Zusman D. R. & Shi W. Y. Type IV pilus of *Myxococcus xanthus* is a motility apparatus controlled by the frz chemosensory system. Curr. Biol. 10, 1143–1146 (2000). 10996798 10.1016/s0960-9822(00)00705-3

[b11] Costerton J. W. . Bacterial biofilms in nature and disease. Annu. Rev. Microbiol. 41, 435–464 (1987). 3318676 10.1146/annurev.mi.41.100187.002251

[b12] Conrad J. C. . Flagella and pili-mediated near-surface single-cell motility mechanisms in *P. aeruginosa*. Biophys. J. 100, 1608–1616 (2011). 21463573 10.1016/j.bpj.2011.02.020 PMC3072661

[b13] Gibiansky M. L. . Bacteria use type IV pili to walk upright and detach from surfaces. Science 330, 197–U150 (2010). 20929769 10.1126/science.1194238

[b14] Jin F., Conrad J. C., Gibiansky M. L. & Wong G. C. L. Bacteria use type-IV pili to slingshot on surfaces. Proc. Natl Acad. Sci. USA 108, 12617–12622 (2011). 21768344 10.1073/pnas.1105073108 PMC3150923

[b15] Stoodley P., Sauer K., Davies D. G. & Costerton J. W. Biofilms as complex differentiated communities. Annu. Rev. Microbiol. 56, 187–209 (2002). 12142477 10.1146/annurev.micro.56.012302.160705

[b16] Nudleman E. & Kaiser D. Pulling together with type IV pili. J. Mol. Microbiol. Biotechnol. 7, 52–62 (2004). 15170403 10.1159/000077869

[b17] Whitchurch C. B. . Characterization of a complex chemosensory signal transduction system which controls twitching motility in *Pseudomonas aeruginosa*. Mol. Microbiol. 52, 873–893 (2004). 15101991 10.1111/j.1365-2958.2004.04026.x

[b18] Trampuz A. & Zimmerli W. Diagnosis and treatment of infections associated with fracture-fixation devices. Injury 37, 59–66 (2006). 10.1016/j.injury.2006.04.010 16651073

[b19] Yeung A. T. Y., Parayno A. & Hancock R. E. W. Mucin promotes rapid surface motility in *Pseudomonas aeruginosa*. mBio 3, e00073–12 (2012). 22550036 10.1128/mBio.00073-12 PMC3569861

[b20] Kamatkar N. G. & Shrout J. D. Surface hardness impairment of quorum sensing and swarming for *Pseudomonas aeruginosa*. PLoS ONE 6, e20888 (2011). 21687741 10.1371/journal.pone.0020888 PMC3110244

[b21] Suzuki H. . Precise synthesis and physicochemical properties of high-density polymer brushes designed with poly(N-isopropylacrylamide). Macromolecules 43, 9945–9956 (2010).

[b22] Liu G. M. & Zhang G. Z. Collapse and swelling of thermally sensitive Poly(N-isopropylacrylamide) brushes monitored with a quartz crystal microbalance. J. Phys. Chem. B 109, 743–747 (2005). 16866436 10.1021/jp046903m

[b23] Wang X. H., Qiu X. P. & Wu C. Comparison of the coil-to-globule and the globule-to-coil transitions of a single poly(N-isopropylacrylamide) homopolymer chain in water. Macromolecules 31, 2972–2976 (1998).

[b24] Voinova M. V., Rodahl M., Jonson M. & Kasemo B. Viscoelastic acoustic response of layered polymer films at fluid-solid interfaces: continuum mechanics approach. Phys. Scr. 59, 391–396 (1999).

[b25] Ishida N. & Kobayashi M. Interaction forces measured between poly(N-isopropylacrylamide) grafted surface and hydrophobic particle. J. Colloid. Interf. Sci. 297, 513–519 (2006). 10.1016/j.jcis.2005.10.068 16343523

[b26] Netz R. R. & Andelman D. Neutral and charged polymers at interfaces. Phys. Rep. 380, 1–95 (2003).

[b27] Donoho D. L. & Johnstone I. M. Ideal denoising in an orthonormal basis chosen from a library of bases. Compt. Rend. Acad. Sci. Paris Ser. A 319, 1317–1322 (1994).

[b28] Deziel E., Lepine F., Milot S. & Villemur R. rhlA is required for the production of a novel biosurfactant promoting swarming motility in *Pseudomonas aeruginosa*: 3-(3-hydroxyalkanoyloxy)alkanoic acids (HAAs), the precursors of rhamnolipids. Microbiology-Sgm 149, 2005–2013 (2003). 10.1099/mic.0.26154-0 12904540

[b29] Lu H. . Microfluidic shear devices for quantitative analysis of cell adhesion. Anal.Chem. 76, 5257–5264 (2004). 15362881 10.1021/ac049837t

[b30] Spirin L. . Polymer-brush lubrication in the limit of strong compression. Eur. Phys. J. E 33, 307–311 (2010). 21107880 10.1140/epje/i2010-10674-3

[b31] Binder K., Kreer T. & Milchev A. Polymer brushes under flow and in other out-of-equilibrium conditions. Soft Matter 7, 7159–7172 (2011).

[b32] Zhao K. . Psl trails guide exploration and microcolony formation in *Pseudomonas aeruginosa* biofilms. Nature 497, 388–391 (2013). 23657259 10.1038/nature12155 PMC4109411

[b33] Wloka M., Rehage H., Flemming H. C. & Wingender J. Rheological properties of viscoelastic biofilm extracellular polymeric substances and comparison to the behavior of calcium alginate gels. Colloid. Polym. Sci. 282, 1067–1076 (2004).

[b34] Queffelec J., Gaynor S. G. & Matyjaszewski K. Optimization of atom transfer radical polymerization using Cu(I)/Tris(2-(dimethylamino)ethyl)amine as a catalyst. Macromolecules 33, 8629–8639 (2000).

[b35] Kizhakkedathu J. N., Norris-Jones R. & Brooks D. E. Synthesis of well-defined environmentally responsive polymer brushes by aqueous ATRP. Macromolecules 37, 734–743 (2004).

[b36] Yim H. . Effects of grafting density and molecular weight on the temperature-dependent conformational change of poly(N-isopropylacrylamide) grafted chains in water. Macromolecules 39, 3420–3426 (2006).

[b37] Wang S. & Zhu Y. Facile method to prepare smooth and homogeneous polymer brush surfaces of varied brush thickness and grafting density. Langmuir 25, 13448–13455 (2009). 19863074 10.1021/la901785t

[b38] Kubota K., Fujishige S. & Ando I. Solution properties of poly(N-isopropylacrylamide) in water. Polym. J. 22, 15–20 (1990).

[b39] Fujishige S. Intrinsic viscosity-molecular weight relationships for poly(N-isopropylacrylamide) solutions. Polym. J. 19, 297–300 (1987).

[b40] Schorr P. A., Kwan T. C. B., Kilbey S. M., Shaqfeh E. S. G. & Tirrell M. Shear forces between tethered polymer chains as a function of compression, sliding velocity, and solvent quality. Macromolecules 36, 389–398 (2003).

[b41] Graessley W. W., Masuda T., Roovers J. E. L. & Hadjichristidis N. Rheological properties of linear and branched polyisoprene. Macromolecules 9, 127–141 (1976).

[b42] Tadmor R., Janik J., Klein J. & Fetters L. J. Sliding friction with polymer brushes. Phys. Rev. Lett. 91, 115503 (2003). 14525437 10.1103/PhysRevLett.91.115503

[b43] Heydorn A. . Experimental reproducibility in flow-chamber biofilms. Microbiology 146, 2409–2415 (2000). 11021917 10.1099/00221287-146-10-2409

