# Supplementary material for: Bacteria slingshot more on soft surfaces
Source: Nat Commun. 2014 Nov 21;5:5541. doi: 10.1038/ncomms6541 (PMC4263163; doi:10.1038/ncomms6541)
Supplement: Supplementary Information — Supplementary Figures 1-8, Supplementary Table 1, Supplementary Note 1, Supplementary Methods and Supplementary References. [file ncomms6541-s1.pdf]

Supplementary Figure 1

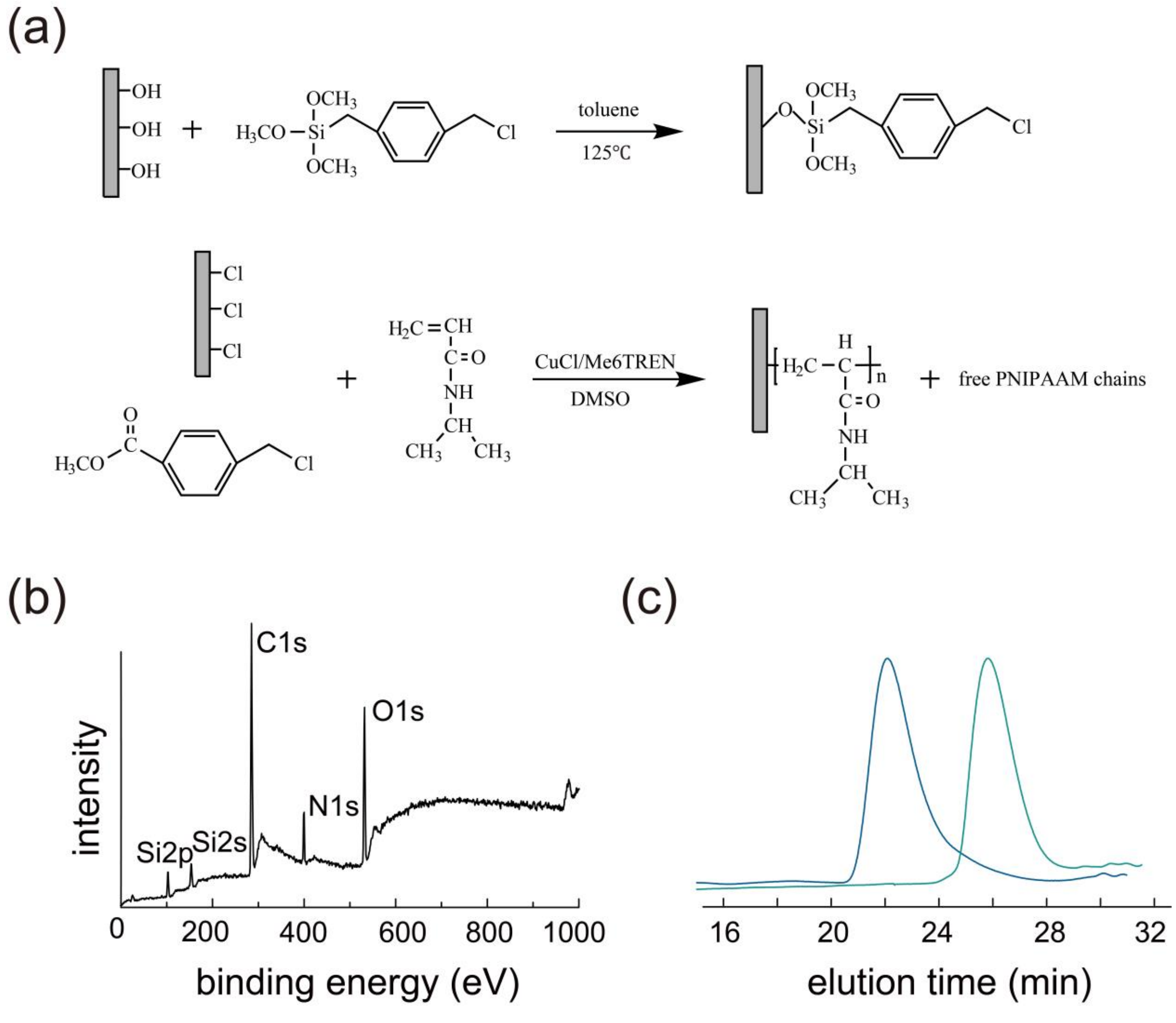

Supplementary Figure 1. Preparation and Characterization of PNIPAAM brush surfaces (*a*) Schematic showing that the method was used to graft PNIPAAM chains on coverslips. (*b*) Representative X-ray photoelectron spectrum of PNIPAAM brush surfaces. (*c*) Elution times of PNIPAAM chains that were polymerized in the solutions, where green or blue line arose from the free polymer chains collecting from the reactions for preparation of

short or long-brush surfaces respectively.

Supplementary Figure 2

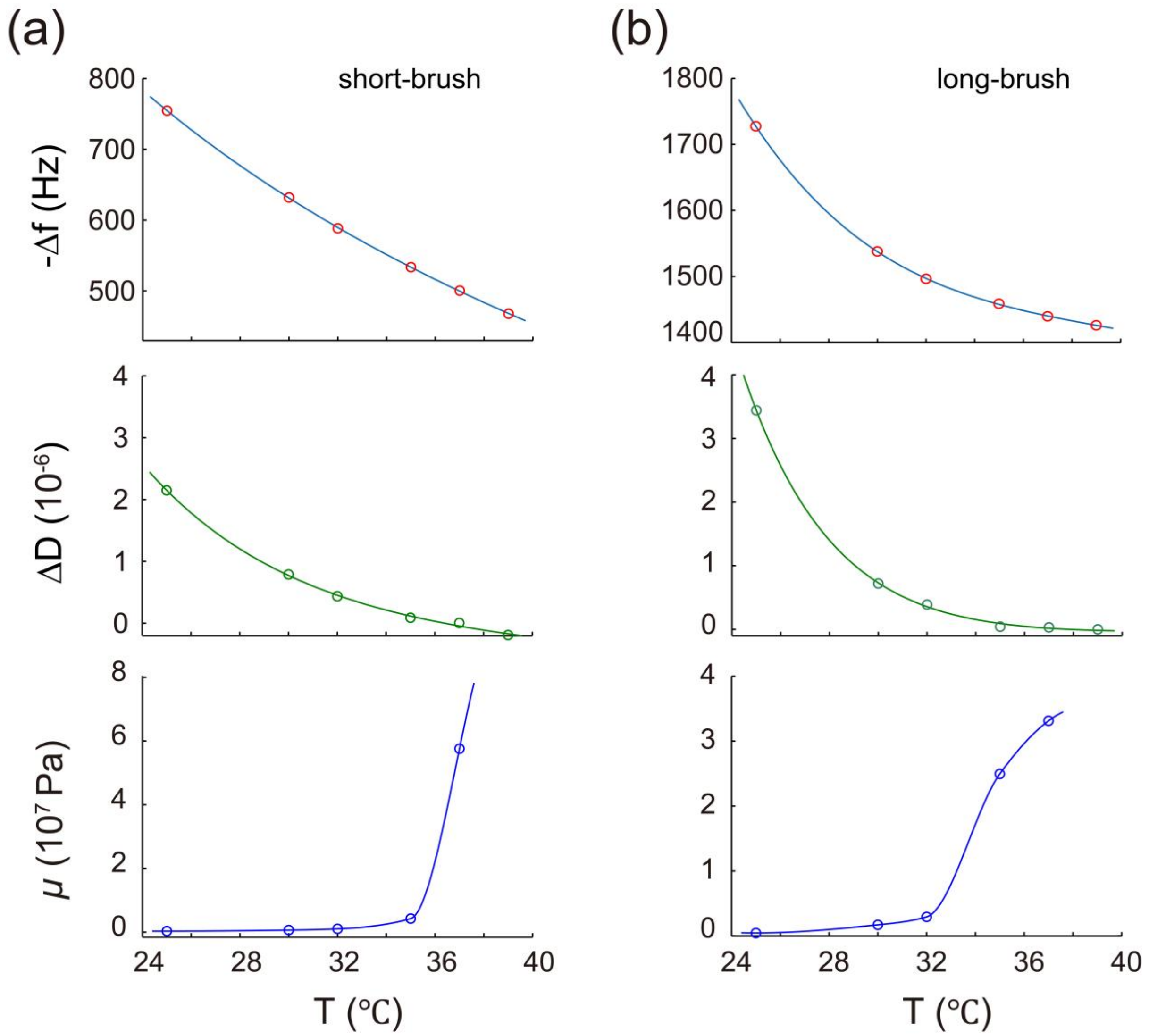

Supplementary Figure 2. Characterization of the viscoelasticity of PNIPAAM brush surfaces at different temperatures Temperature dependences of the frequency or the dissipation shift ($-\Delta f$ or ΔD) as well as the shear modules (μ) of (a) short-brush or (b) long-brush surfaces.

Supplementary Figure 3

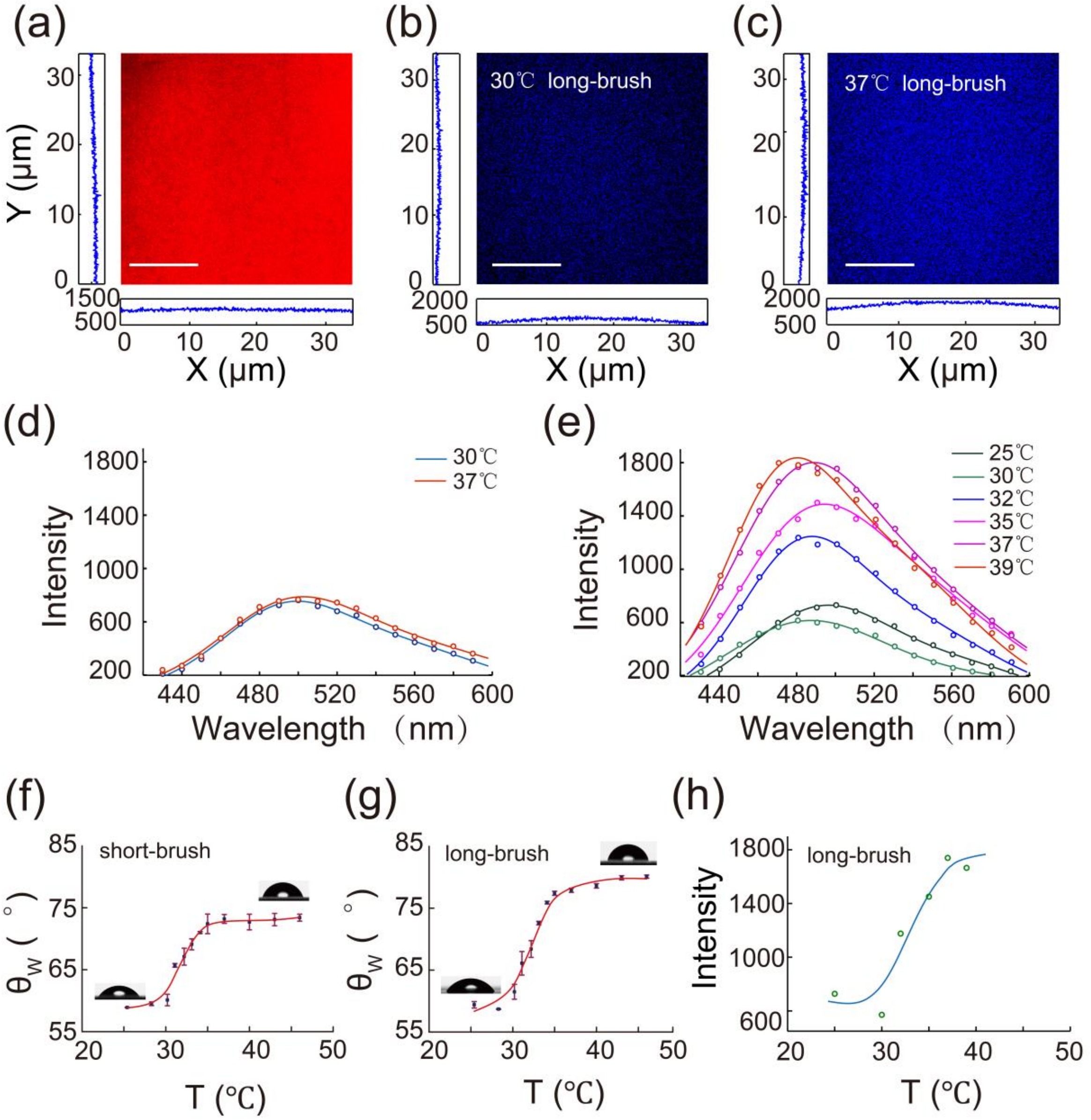

Supplementary Figure 3. Characterization of the surface heterogeneity of PNIPAAM brush surfaces Representative fluorescence images for (*a*) rhodamine-B labeled (30 ℃), (*b*) ANS labeled (30 ℃) and (*c*) ANS labeled (37 ℃) long-brush surfaces, where scale bars for all images are 10 μm. Temperature dependences of the fluorescence spectrum for (*d*) ANS

labeled glass surfaces or (*e*) ANS labeled long-brush surfaces. Temperature dependences of the contact angle on (*f*) short-brush or (*g*) long-brush surfaces. (*h*) Temperature dependences of fluorescent intensities at 500 nm on long-brush surfaces. The error bars in the *(f)* and *(h)* represent standard deviations arising from ≥3 technical replicates.

Supplementary Figure 4

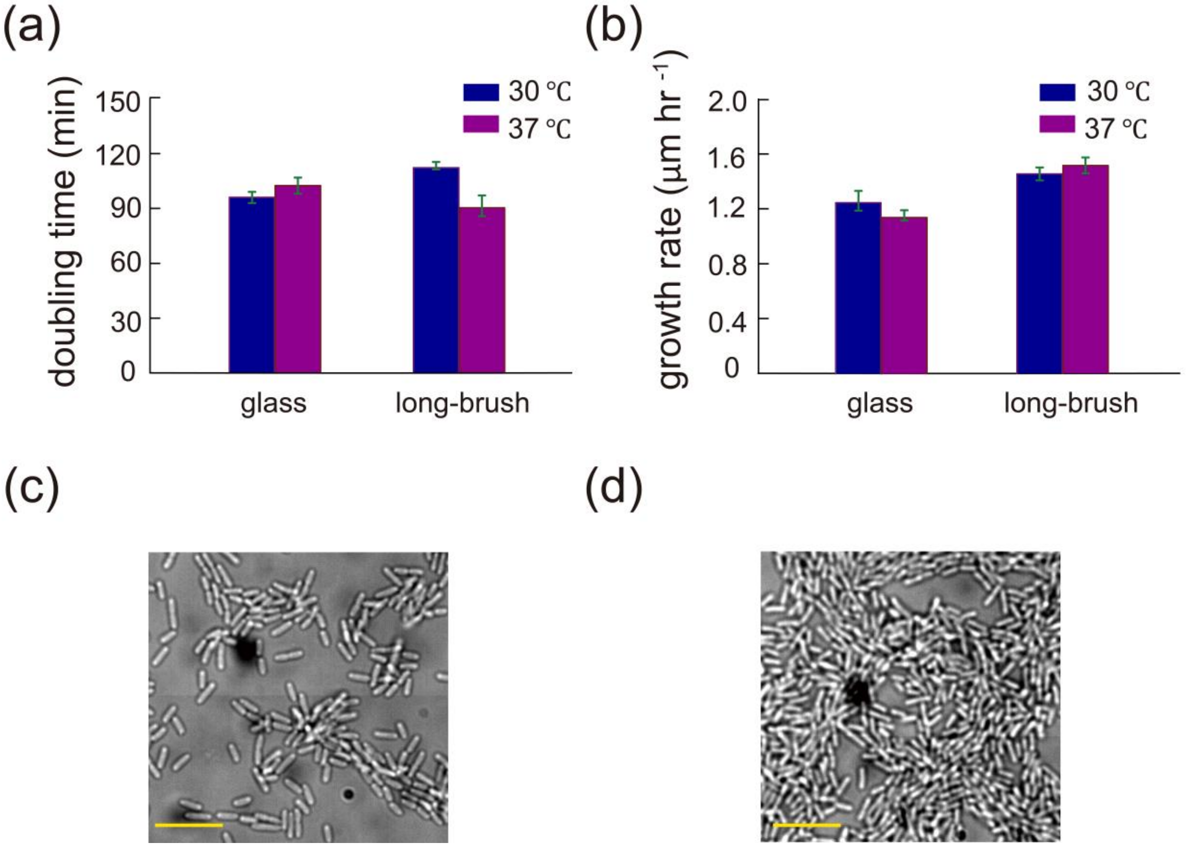

Supplementary Figure 4. Growth of *P. aeruginosa* on PNIPAAM brush surfaces *(a)* Doubling time or (*b*) growth rate of *P*. *aeruginosa* grown on glass or long-brush surfaces, where the average doubling time or the growth rate in each condition arose from the analysis of multiple cells (≥40), the results obtained from multiple technical replicates (≥3) and the error bar represents a standard deviation. Representative bright-field images show that the growth of *P*. *aeruginosa* on long-brush surfaces after (*c*) 12 or (*d*) 17 hours, where scale bars for all images are 10 μm.

Supplementary Figure 5

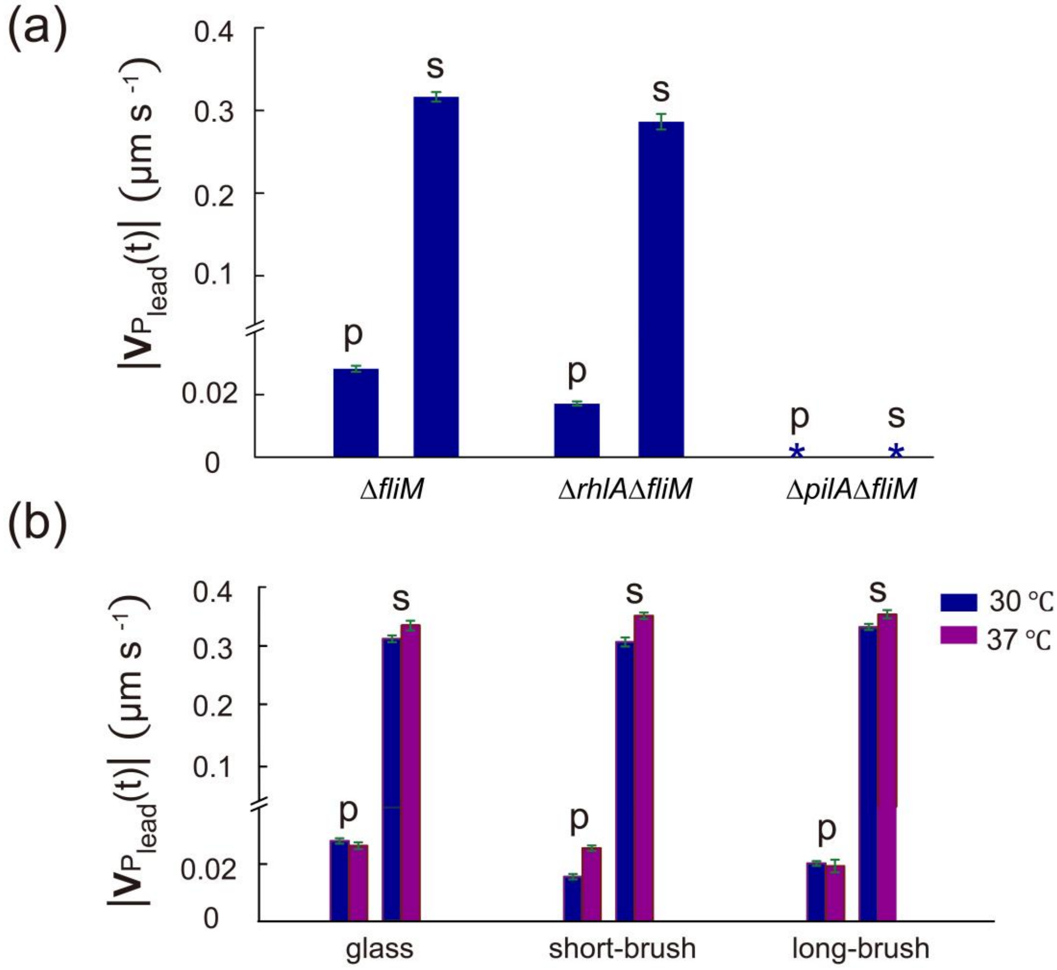

Supplementary Figure 5. Crawling motility of *P. aeruginosa* on different surfaces Crawling motility of *(a)* different mutants of *P. aeruginosa* on glass surfaces at 30 ℃ or (*b*) $\Delta fliM$ mutant on different surfaces at 30 ℃ or at 37 ℃, where the symbol of "p" or "s" represents pulling actions or slingshots respectively, "*" represents no detectable motions, the average velocities in each condition arose from the analysis of multiple cells (≥50), the results obtained from multiple technical replicates (≥3) and the error bar represents a

standard deviation.

Supplementary Figure 6

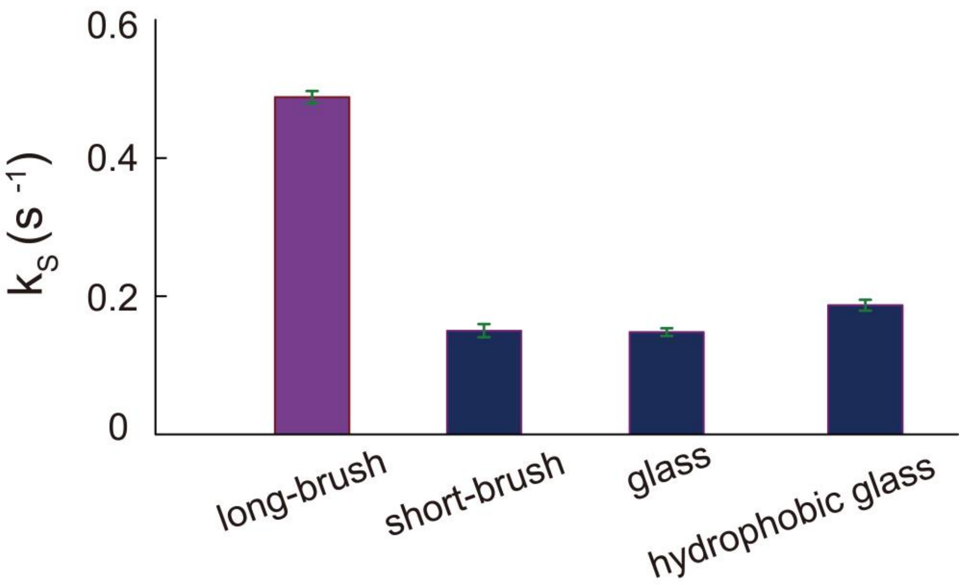

Supplementary Figure 6. Slingshot of *P. aeruginosa* on hydrophobic surfaces Slingshot of *P. aeruginosa* on different surfaces at 30 ℃, where the average rate of slingshots in each condition arose from the analysis of multiple cells (35 to 77), the results obtained from multiple technical replicates (≥3) and the error bar represents a standard deviation.

Supplementary Figure 7

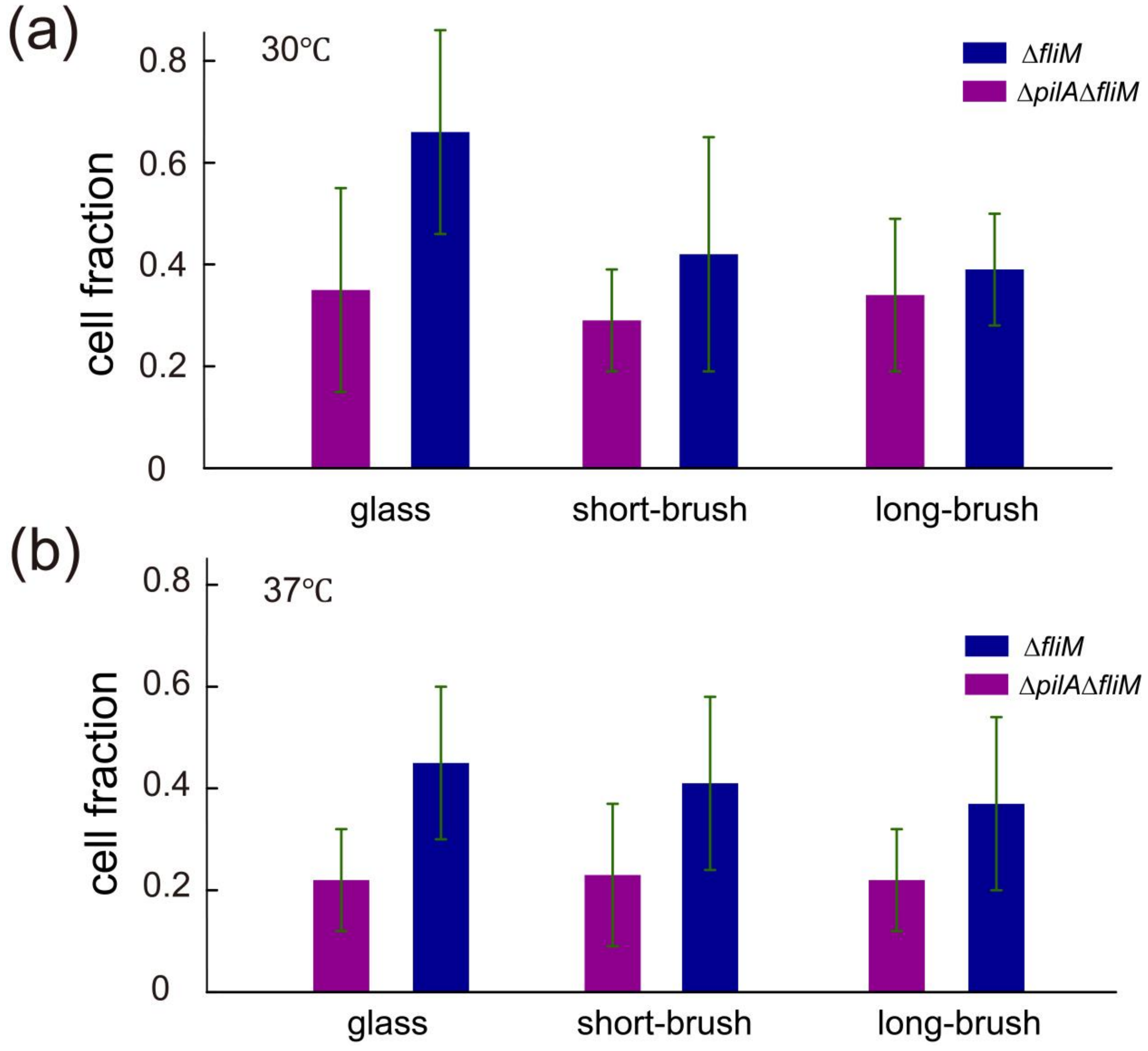

Supplementary Figure 7. Evaluation of the interactions between *P. aeruginosa* and surfaces Retentions of different *P. aeruginosa* mutants on glass, short or long-brush surfaces at (*a*) 30 ℃ or at (*b*) 37 ℃, where the average retentions in each condition arose from the analysis of multiple cells (≥200), the results obtained from multiple technical replicates (≥3)

and the error bar represents a standard deviation.

Supplementary Figure 8

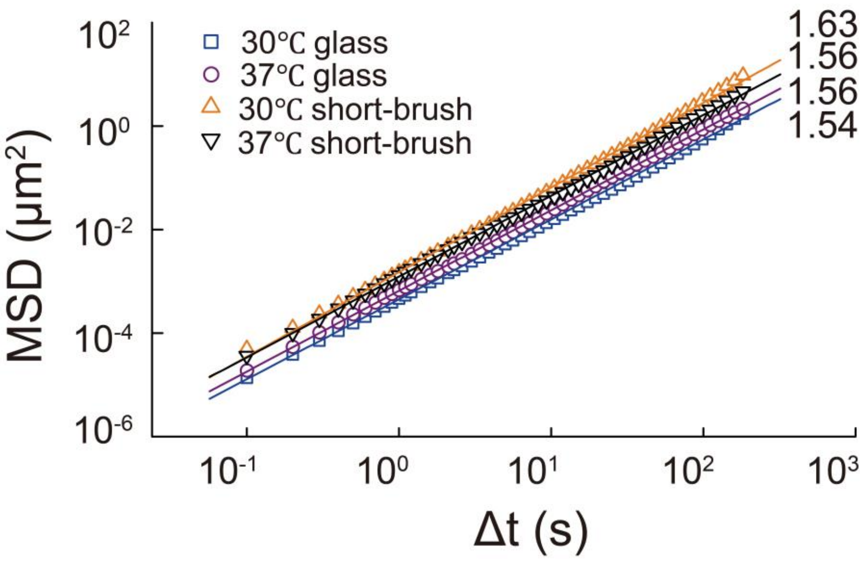

Supplementary Figure 8. Crawling trajectories of *P. aeruginosa* cells in control experiments Representative mean square displacement (MSD) of the trajectory on unmodified coverslips or short-brush surfaces at 30 ℃ or at 37 ℃.

Supplementary Table 1

	Description	Source
**Strains**		
PAO1bΔ*fliM*	non-flagellated *P. aeruginosa* ATCC 15692 strain	J.D. Shrout
PAO1bΔ*pilA*	non-piliated *P. aeruginosa* ATCC 15692	J.D. Shrout
PAO1b Δ*rhlA*Δ*fliM*	*rhlA*::Gm^r derivative of non-flagellated *P. aeruginosa fliM*::Gm^r	This study
PAO1b Δ*pilA* Δ*fliM*	derivative of non-piliated *P. aeruginosa*	This study
**Plasmids**		
pEX18Ap	Ap^r; gene replacement vector with MCS from pUC18	H.P. Schweizer
pFGM1	Ap^r, Gm^r ; source of gentamicin *FRT -aacC1 -FRT* cassette	H.P. Schweizer
pEX18Ap-rhlA	Ap^r; in-frame deletion of *rhlA* constructed by PCR and cloned into EcoRI/HindIII sites of pEX18Ap	This study
pEX18Ap-rhlAGm	Ap^r, Gm^r; PstI fragment from pFGM1 containing the gentamicin-resistant cassette cloned into PstI site of pEX18Ap-rhlA	This study
pEX18Ap-fliM	Ap^r; in-frame deletion of *fliM* constructed by PCR and cloned into EcoRI/HindIII sites of pEX18Ap	This study
pEX18Ap-fliMGm	Ap^r, Gmr; PstI fragment from pFGM1 containing the gentamicin-resistant cassette cloned into PstI site of pEX18Ap-fliM	This study
**Primers Sequence**		
rhlAupEF	5'-GAGATGAATTCCTCGTAGACCGGCTCGATCAT-3'	
rhlAupPR	5'-GAGATCTGCAGATGGCCATCGGCTACGCCTGA-3'	
rhlAdnPF	5'-GAGATCTGCAGCAACAGACTTTCGCGCCGCAT-3'	
rhlAdnHR	5'-GAGATAAGCTTGAGGCCTGCGAAGTGTCCTAT-3'	
fliMupEF	5'-GTATGAATTCTCCAGCCAGAACCTGAAGTC-3'	
fliMupXR	5'-GTATTCTAGAGATTTCATCCTGGGAAAG-3'	
fliMdnXF	5'-GTATTCTAGAACCTGGCGCTACAGATTCTC-3'	
fliMdnHR	5'-GTATAAGCTTTGGCTGTTGATCTTGTCGAA-3'	

Supplementary Table1 Strains, plasmids, and primers used in this study.

Supplementary Note 1

Estimation of the longest relaxation time of PNIPAAM brush surfaces According to the scaling theory, the longest relaxation time (τ) of a concentered polymer solution (linear or star polymer) is proportional to $\xi(c)(cM)^{\alpha}(\rho M'/cM)(M/ck_BT)$, where $\xi(c)$ is the concentration (c) dependent friction coefficient, M, M' or ρ is the molecular weight, characteristic molecular weight or bulk density of the polymer, α is the scaling exponent, k_BT is the thermal energy (1). By applying the relations of $\xi(c) \propto c^{1.5}$ and $\alpha = 2.0$ (2), τ can be further expressed as

$$\tau \propto c^{1.5}M^{2.0} \quad (1)$$

Graesslery *et. al*. have shown that τ is around ~200 seconds for a 4-arms poly(isoprene) concentrated solution with $M_w = 1.96 \times 10^6\ g \cdot mol^{-1}$ and $c = 0.257\ g \cdot mol^{-1}$ (1). Using the Eq. (1), one can find that τ is around 0.08 or 8 seconds for a short or long star-polymer solution respectively, where M_w and c are set to be identical to that of the short or long PNIPAAM grafting chains. Further studies suggested that τ would be even longer on brush surfaces than that in concentrated star-polymer solutions with a enhance factor up to 10 (3); Taken together, the magnitudes of τ with one order uncertainty were

estimated for short or long-brush surfaces, i.e., $\tau_{short} = 0.1 \sim 1$s and τ_{long} =10 ~ 100 s.

Supplementary Methods

Construction of the *P. aeruginosa* mutants Two mutants, $\Delta rhlA\Delta fliM$ and $\Delta pilA\Delta fliM$, were constructed for this study. The upstream region of *rhlA* (380bp) was amplified by PCR using primers rhlAupEF (with EcoRI site) and rhlAupPR (with PstI site), while the downstream region (540bp) was amplified with primers rhlAdnPF (with PstI site) and rhlAdnHR (with HindIII site) (Supplementary Table 1). After digestion by EcoRI/PstI or PstI/HindIII, respectively, the two DNA fragments were cloned into a gene replacement vector pEX18Ap between EcoRI and HindIII restriction sites via a three-piece ligation. Next, the constructed plasmid pEX18Ap-rhlA was digested with PstI and then ligated with a gentamicin-resistant (Gm^r) cassette, which was obtained from pFGM1 plasmid by digesting with PstI. The constructed plasmid (pEX18Ap-rhlAGm) was electroporated into PAO1b$-\Delta fliM$ as described previously (4). The recombinant strain $\Delta rhlA\Delta fliM$ was identified by screening on LB agar plates containing 5% (w/v) sucrose with 30 $\mu g \cdot mL^{-1}$ gentamicin. The $\Delta pilA\Delta fliM$ mutant was constructed by transforming plasmid pEX18Ap-fliMGm into PAO1b$-\Delta pilA$ using the same method. Mutants of $\Delta rhlA\Delta fliM$ and $\Delta pilA\Delta fliM$ were finally verified by PCR and sequencing.

Characterization of the viscoelasticity of PNIPAAM brush surfaces at different

temperatures PNIPAAM brushes with distinctive molecular weights (denoting by short or long-brush below) were grafted on the SiO_2-coated crystal surfaces using a method that was completely identical to that for grafting PNIPAAM brushes on the glass coverslips (see Methods in the main text), which ensures that the molecular weights and the grafting-densities of PNIPAAM chains grafted on the SiO_2-coated crystal surfaces were comparable to that grafted on the glass surfaces. Next, we directly measured the frequency and the dissipation shift (Δf and ΔD) of *short* or *long* PNIPAAM brush surfaces immersed in FAB mediums at different temperatures (T) using a quartz crystal microbalance equipped with dissipation monitoring unit (QCM-D), in which an AT-cut quartz crystal (from Q-sense AB) with a fundamental resonant frequency of 5 MHz was used, Δf is measurable to within ± 1 Hz in aqueous mediums and T was controlled in the range of ± 0.02 ℃ (5). Our results clearly indicate that regardless the molecular weights of grafting chains raising T from 25 ℃ to 40 ℃ leads to the decreasing of $-\Delta f$ and ΔD, as shown in Supplementary Fig. 2, reflecting that the hardness of the brush surfaces would increase companying the collapsing of grafting chains. These results are consistent with the literature report (5).

To further quantify the viscoelasticity of PNIPAAM brush surfaces at different temperatures, a model (Voigt) was applied to interpret the temperature dependences of $\Delta f(T)$ and $\Delta D(T)$, where a viscoelastic polymeric film with a uniform thickness immersed in a semi-infinite Newtonian fluid under a no-slip condition were assumed (6). According this model and using a thin-layer approximation, $-\Delta f$ and ΔD can be expressed as,

$$\Delta f \simeq -\frac{1}{2\pi\rho_0 h_0}[h_{eq}\rho_b\omega - 2h_{eq}(\frac{\eta_m}{\delta_m})^2\frac{\omega^2\eta(\omega)}{\mu^2+\omega^2\eta^2(\omega)}] \quad (2)$$

$$\Delta D \simeq \frac{1}{2\pi f\rho_0 h_0}[2h_{eq}(\frac{\eta_m}{\delta_m})^2\frac{\omega\mu}{\mu^2+\omega^2\eta^2(\omega)}] \quad (3)$$

where ρ_0, h_0, f or ω is the density, thickness, resonant frequency or angular frequency of the quartz resonator respectively, η_m or ρ_m is the viscosity or density of the medium, $\delta_m = (2\eta_m/\omega\rho_m)^{1/2}$, h_{eq} or ρ_b is the thickness or bulk density of the PNIPAAM brush layer, μ or $\eta(\omega)$ is the shear modulus or shear viscosity of the brush layer (6). Combining of Eqs.2 and 3, one can find that μ can be further expressed as,

$$\mu \simeq \eta_m(\frac{\rho_m}{\rho_b})(\frac{\Delta f}{\Delta D}) \quad (4)$$

at the condition of $\mu/\,\omega\eta(\omega) \gg 1$; note that $\mu/\,\omega\eta(\omega)$ is in the range of 10^1 to 10^3 in our experiments. By applying Eq. 4, we found that raising T from 25 ℃ to 40 ℃ leads to the increasing of μ (~ 40 to 100 folds) significantly, as shown in Supplementary Fig. 2, which directly demonstrate that thermosensitive PNIPAAM brush surfaces could switch from soft $(\mu \sim 10^5 Pa)$ to hard $(\mu \sim 10^7 Pa)$ by a small temperature change; note that at $T < 32$ ℃, the magnitude of μ is comparable to that of a non-thermosensitive protein film (7). Furthermore, one can estimate the magnitude of $\eta(\omega)$ using Eqs. 2 and 4, however, the relative error is quite larger due to $\mu/\,\omega\eta(\omega) \gg 1$. We found that the magnitude of $\eta(\omega)$ is around ~$10^{-3} Pa\cdot s$ at $T = 30$ ℃, which is much smaller than its corresponded static viscosity $\eta(\omega = 0)$ (10^{-1} to $10^0 Pa\cdot s$), clearly indicating that PNIPAMM brush surfaces

are shear-thinning at $T = 30$ ℃.

Characterization of the spatial distribution of grafting chains on long-brush surfaces To directly visualize the localization of long grafting chains on surfaces, we grafted the PNIPAAM chains on the coverslips using SI-ATRP, in which a trace amount of methacryloxyethyl thiocarbamoyl rhodamine-B (MRB, Polysciences) was added to copolymerize with NIPAAM. The synthetic method is completely identical to that for preparing non-labeled PNIPAAM long-brush surfaces (see Methods in the main text), except MRBs with a weight ratio of 1:2500 (MRB:NIPAAM) were added in the SI-ATRP reaction, which ensure that the molecular weight or grafting-density of labeled chains is comparable to that of non-labeled chains. Next, a fluorescent microscope (IX81, Olympus) equipped with a $100\times$ oil objective was used to visualize the surface localizations of grafting chains on coverslips immersed in FAB medium at 30 ℃. Rhodamine-B labeled grafting chains was excited with a 565 ± 25nm LED illuminator and the resulting fluorescence was collected through an emission filter (600 ± 25nm). Images of dimension 512 pixels × 512 pixels were captured by a sCMOS camera (Neo, Andor). We found that the fluorescent intensities in the acquired images are rather even, a representative image as shown in Supplementary Fig. 3*a*, displaying that the spatial distribution of those grafting chains are homogenous on surfaces at the length scale ranging from sub-micron to tens of microns. This result further demonstrate that PNIPAAM chains can be homogeneously grafted on the coverslips using SI-ATRP.

Characterization of the local hydrophobicity on long-brush surfaces at different

temperatures To explore the local hydrophobicity on long-brush surfaces at different temperatures, we labeled long-brush surfaces with the 8-Anilino-1-naphthalenesulfonic (ANS, Sigma) because the fluorescent intensities of ANS depend on the hydrophobicity of local environments that they met (8). The labeling procedures are as follows: ANS were directly dissolved in FAB mediums to a concentration of 100 mM; the resultant labeling buffer was injected into flow-cells and allowed to rest for 5 hours to wait ANS to adsorb to the long-brush surfaces. Next, a confocal microscope equipped spectroscopical unit (FluoView FV1000, Olympus) was used to collect the fluorescence spectrum images of ANS adsorbed on long-brush at different temperatures, in which ANS was excited with a 405 nm laser and the resulting fluorescence spectrums were collected through a scanning grating with a scan range from 420 to 590 nm (10 nm per step) and a bandwidth of 2 nm. Spectrum images of dimension 512 pixels × 512 pixels were scanned and recorded by a photomultiplier (PMT).

The fluorescence spectrums of ANS adsorbed on glass surfaces were collected either at 30 ℃ or at 37 ℃ and then were used for negative controls, as shown in Supplementary Fig. 3*d*. We found that changing temperatures does not affect the fluorescence spectrums of ANS adsorbed on glass surfaces, indicating that hydrophobicity of glass surfaces are nearly independent of temperatures. By contrast, we found that raising T from 30 ℃ to 37 ℃ leads to the increasing of the emissions of ANS adsorbed on long-brush PNIPAAM surfaces significantly, as shown in Supplementary Fig. 3*e*. These results clearly indicate that the hydrophobicity of long-brush surfaces increased as the increasing of temperature, as shown in Supplementary Fig, 3*h*, consisting with the results arising from the measurements

of contact angles (Supplementary Fig. 3g). Most importantly, we found that the spectrum images of ANS are rather even at different temperatures, where the representative image collected at $30\ ℃$ or at $37\ ℃$ was shown in Supplementary Fig. 3b or c. Note that the slightly dim fluorescence in the edges at Supplementary Fig. 3b or c arose from uneven illuminations. These results demonstrate that the local hydrophobicity are homogenous on the long-brush surfaces at different temperatures in the length scale ranging from sub-micron to tens of microns.

Evaluation of the interactions between *P. aeruginosa* and surfaces Polydimethylsiloxane (PDMS) microfluidic channels with a dimension of $300\ \mu m \times 20\ \mu m$ (width × height or $w \times h$) were fabricated using soft lithography techniques (9). Next, we directly evaluated the interactions between bacteria and surfaces using surface-shear stress assays (10, 11). In the surface-shear stress assays, 400 μL bacterial cultures, harvesting at an exponential phase, were diluted in the 600 μL fresh FAB mediums. The resultant cultures were injected into PDMS channels and allowed to rest for 15 minutes to allow bacteria to attach to the coverslip, after which unattached cells were washed out gently. A flow with a flow rate (Q) of $7.0\ mL \cdot hr^{-1}$ was applied to shear these surface-attached cells to enforce them detaching from surfaces, where the shear stress ($\sigma \simeq 78\ Pa$ or $78\ pN \cdot \mu m^{-2}$) can be calculated using a relation $\sigma \simeq 6Q\eta_m/wh^2$ (11). Meanwhile, an inverted microscope (IX81, Olympus) equipped with a $100\times$ oil objective was used to record the number of undetached cells in the first 30 minutes. This assay was replicated 4 times in each identical condition. Finally, the percentage of undetached cells were used to evaluate the interactions between bacteria and

surfaces due to the fact that stronger cell-surface interactions lead to less detachments.

To further investigate the interactions solely arising from bacterial body and surfaces, we constructed a mutant ($\Delta pilA\Delta fliM$) that lacks both surface appendages of TFP and flagellum. Our results clearly indicate that $\Delta fliM$ cells remain more on glass surfaces either at 30 ℃ or at 37 ℃ comparing to the retentions of $\Delta pilA\Delta fliM$ mutant, as shown in Supplementary Fig.7, which indicates that TFP could facilitate adhesion of cells on glass surfaces, consisting with the literature report (12). Comparing to the results arising from $\Delta fliM$ or $\Delta pilA\Delta fliM$ cells on glass surfaces at 30 ℃, we found that the retentions of $\Delta fliM$ cells were suppressed slightly on PNIPAAM brush surfaces, whereas the retentions of $\Delta pilA\Delta fliM$ cells were nearly unchanged. These results suggest that the adhesions between TFP and PNIPAAM brush surfaces are relative weaker comparing to that between TFP and glass surfaces. Most importantly, we found that the retention of $\Delta fliM$ or $\Delta pilA\Delta fliM$ mutant on the short-brush is almost identical to that on the long-brush surfaces either at 30 ℃ or at 37 ℃, demonstrating that the interactions between bacteria and short or long-brush surfaces are nearly identical. Therefore, we concluded that using short-brush surfaces as negative controls is sufficient to exclude the fact that slingshot more on long-brush surfaces arose from different interactions between bacteria and surfaces.

Supplementary References

1. Graessley, W. W., Masuda, T., Roovers, J. E. L. & Hadjichristidis, N. Rheological properties of linear and branched polyisoprene. *Macromolecules* **9**, 127-141 (1976).
2. Schorr, P. A., Kwan, T. C. B., Kilbey, S. M., Shaqfeh, E. S. G. & Tirrell, M. Shear forces between tethered polymer chains as a function of compression, sliding velocity, and solvent quality. *Macromolecules* **36**, 389-398 (2003).
3. Tadmor, R., Janik, J., Klein, J. & Fetters, L. J. Sliding friction with polymer brushes. *Phys. Rev. Lett.* **91**, 115503 (2003).
4. Choi, K. H., Kumar, A. & Schweizer, H. P. A 10-min method for preparation of highly electrocompetent Pseudomonas aeruginosa cells: Application for DNA fragment transfer between chromosomes and plasmid transformation. *J. Microbiol. Methods* **64**, 391-397 (2006).
5. Liu, G. M. & Zhang, G. Z. Collapse and swelling of thermally sensitive Poly(N-isopropylacrylamide) brushes monitored with a quartz crystal microbalance. *J. Phys. Chem. B* **109**, 743-747 (2005).
6. Voinova, M. V., Rodahl, M., Jonson, M. & Kasemo, B. Viscoelastic acoustic response of layered polymer films at fluid-solid interfaces: Continuum mechanics approach. *Phys. Scr.* **59**, 391-396 (1999).
7. Hook, F., *et al.* Variations in coupled water, viscoelastic properties, and film thickness of a Mefp-1 protein film during adsorption and cross-linking: A quartz crystal microbalance with dissipation monitoring, ellipsometry, and surface plasmon

resonance study. *Anal. Chem.* **73**, 5796-5804 (2001).

8. Ye, X. D., *et al.* How many stages in the coil-to-globule transition of linear homopolymer chains in a dilute solution? *Macromolecules* **40**, 4750-4752 (2007).

9. Qin, D., Xia, Y. N. & Whitesides, G. M. Soft lithography for micro- and nanoscale patterning. *Nat. Protoc.* **5**, 491-502 (2010).

10. Lu, H., *et al.* Microfluidic shear devices for quantitative analysis of cell adhesion. *Anal. Chem.* **76**, 5257-5264 (2004).

11. Shen, Y., Siryaporn, A., Lecuyer, S., Gitai, Z. & Stone, H. A. Flow Directs Surface-Attached Bacteria to Twitch Upstream. *Biophys. J.* **103**, 146-151 (2012).

12. Jenkins, A. T. A, Buckling, A., McGhee, M. & ffrench-Constant, R. H. Surface plasmon resonance shows that type IV pili are important in surface attachment by Pseudomonas aeruginosa. *J. R. Soc. Interface* **2**, 255-259 (2005).
